# Agricultural Journals

**Published:** 1857-11

**Authors:** 


### AGRICULTURE.

THE healthiest of all callings, and which, when intelligently prosecuted, involves a large share of bodily activities, with a wide range of intellectual and scientific inquiry, deserves more attention than the present age accords to it. One of the greatest mistakes of the times is, that "*anybody has sense enough to be a farmer*," that it is a pursuit which can be taken up and successfully prosecuted without pre-culture. As well might you set a hodman to draft a national capital. With these views, and with a desire to promote *scientific agriculture*, as the basis of national thrift, independence, and wealth, we append a list of Agricultural Periodicals, published regularly, earnestly recommending our "Farm Readers," in every section of the country, to patronize them liberally, always giving the preference to those nearest them.

MAINE.—*Maine Farmer*, Augusta: weekly, folio, 4 pages, $1.75. Russell Eaton, Publisher and Proprietor; Ezekiel Holmes, Editor.

NEW HAMPSHIRE.—*Farmers' Cabinet*, Amherst; weekly, folio, 4 pages, $1.50. E. D. Boylston, Editor and Proprietor.

*Farmer and Visitor*, Manchester; weekly, folio, 4 pages.

*Granite Farm.*—Concord; monthly, quarto, $1.

VERMONT.—*Vermont Stock Journal*, Middlebury; monthly, quarto, 16 pages, 50 cents. D. C. Linsley, Editor, Publisher, and Proprietor.

*Green Mountain Farmer.*—West Randolph; weekly, folio, 4 pages, $1.50. A. Perkins, Jr., Editor.

MASSACHUSETTS.—*Boston Cultivator*, Boston; weekly, quarto, 8 pages, $2. Otis Brewer, Publisher; J. Pedder, S. Howard, and O. Brewer, Editors.

*Hovey's Magazine of Horticulture*, Boston; monthly, octavo, 60 pages, $2. Hovey & Co., Publishers; C. M. Hovey, Editor.

*New England Farmer*, Boston; Simon Brown and Wm. Simonds, Editors. Frederick Holbrook and Henry T. French, Associate Editors. Joel Nourse, Proprietor. Weekly. Terms, $2.

*Massachusetts Plowman*, Boston; W. Buckminister and W. J. Buckminister, Editors. Weekly. Terms, $2.

CONNECTICUT.—*The Homestead*, Hartford; weekly, quarto, 8 pages, $2. M. C. Weld, Publisher; William Clift, T. S. Gold, H. A. Dyer, and C. M. Weld, Editors.

NEW YORK.—*American Agriculturalist*, New York City; monthly, quarto, 24 pages, $1. Orange Judd, Editor and Proprietor.

*Working Farmer*, New York City; monthly, quarto, 24 pages, $1. Fred. McCready, Publisher; J. J. Mapes and Wm. Dodge, Editors.

*Country Gentleman*, Albany; Luther Tucker, Editor and Proprietor. Weekly. Terms, $2.

*Rural American*, Utica; semi-monthly, quarto, $1. T. B. Miner, Editor and Proprietor.

*Journal of the New York State Agricultural Society*, Albany; monthly, octavo, 16 pages, B. P. Johnson, Secretary.

*Plow, Loom, and Anvil*, New York City; J. A. Nash and M. P. Parish, Editors. Monthly Magazine. Terms, $2.

*Moore's Rural New Yorker*, Rochester; D. D. T. Moore, Editor, with an able corps of Assistant Editors. Weekly. Terms, $2.

*Northern Farmer*, Utica; T. B. Miner, Editor and Proprietor. Monthly. Terms, $1.

New Jersey.—*New Jersey Farmer*, Freehold; monthly, octavo, 32 pages, $1. Orin Pharo, Editor and Proprietor.

Pennsylvania.—*American Farmer*, Philadelphia; quarto, 4 pages, monthly, 25 cents a year. An Association of Farmers, Editors; Wm. Wallace, Publisher.

*The Horticulturalist*, Philadelphia; monthly, octavo, 64 pages, $2. Dr. J. J. Smith, Editor; Robert P. Smith, Publisher.

*Farm Journal*, Philadelphia; monthly, octavo, 32 pages, $1. David A. Wells, A. M. Spangler, Editors; S. Emlem & Co., Publishers.

*Germantown Telegraph*, (Family and Agricultural paper.) Weekly, folio, Germantown. By Philip R. Freas. $2.

*American Farmer*, Baltimore, Maryland; monthly, octavo, 60 pages, $1. Sands & Worthington, Publishers.

*Rural Southerner*, Ellicott's Mills, Maryland; weekly, quarto, 4 pages, $1. Richard Edwards, Editor and Proprietor.

*Southern Planter*, Richmond, Va.; monthly, octavo, 64 pages, $2. Ruffin & August, Publishers and Proprietors; E. G. Ruffin, Editor.

*Southern Farmer*, Petersburgh, Va., weekly, quarto, 8 pages, $1. Thos. S. Pleasant, A. C. Morton, Editors.

*The Arator*, Raleigh, N. C.; monthly, octavo, 16 pages, $2.

*Carolina Cultivator*, Raleigh, N. C.; monthly, octavo, 34 pages, $2. William D. Cook, Editor and Publisher.

*American Cotton Planter and Soil of the South*, Montgomery, Alabama; monthly, octavo, 32 pages, $1. Underwood and Cloud, Publishers and Proprietors; N. B. Cloud, Agricultural Editor.

*Southern Agriculturalist*, Columbia, S. Carolina; quarto, monthly, $1. Bol. A. G. Sumner, Editor.

*Southern Cultivator*, Augusta, Ga.; monthly, octavo, 24 pages, $1. Wm. Jones, Publisher and Proprietor; D. Lee and Redmond, Editors.

*Tennessee Farmer and Mechanic*, Nashville, Tenn.; monthly, octavo, 48 pages, $2. Smith, Morgan & Co., Publishers; L. P. Williams, Editor.

*The Western Farm Journal*, Louisville, Ky.; weekly, quarto, 16 pages. James P. Hull & Co., Publishers; D. W. Gallagher, Editor.

*Indiana Farmer*, Richmond, Ind.; quarto, 8 pages, monthly, 50 cents. D. P. Halloway, and W. T. Dennis, Editors; Halloway & Co., Proprietors.

*Valley Farmer*, St. Louis, Mo., and Louisville, Ky.; monthly, octavo, 48 pages, $1. N. J. Coleman, Editor and Publisher, St. Louis; H. P. Byram, Editor and Publisher, Louisivlle.

*The Ohio Farmer*, Cleveland, Ohio; weekly, folio, 4 pages, $2. T. Brown, Editor and Proprietor.

*The Ohio Cultivator*, Columbus; semi-monthly, quarto, 16 pages, $1. S. D. Harris, Editor and Proprietor.

*Ohio Valley Farmer*, Cincinnati, Ohio; monthly, quarto, 16 pages, $1. B. F. Sanford, Editor and Proprietor.

*The Cincinnatus*, Farmers' College, near Cincinnati, Ohio; monthly, octavo, 48 pages, $2. F. G. Carey, President of the Farmers' College, Editor.

*Prairie Farmer*, Chicago, Ill. Charles D. Bragdon, John A. Kennicott, and Robert M. Cox, Editors. John S. Wright, Proprietor. Weekly. Terms, $2.

*Spirit of the Agricultural Press*, West Urbana, Ill.; L. G. Chase and Albert Gore, Editors and Publishers. Weekly. Terms, $2.

*Iowa Farmer*, Mount Pleasant, Iowa; octavo, 16 pages, $1. Duane Wilson & Co

*The Northwestern Farmer*, Dubuque, Iowa; monthly, octavo, 42 pages, $1. Mill & Brayton, Editors and Proprietors.

*Wisconsin Farmer*, Madison, Wis.; monthly, octavo, 48 pages, $1. Powers & Skinner, Publishers.

*Michigan Farmer*, Detroit, Mich.; monthly, octavo, 32 pages, $1. R. F. Johnstone, Editor and Publisher.

*California Farmer*, San Francisco and Sacramento (simultaneously), Cal.; weekly, quarto, 8 pages, $5. Warren & Co., Publishers and Proprietors.

---

What is worth doing at all is worth doing well.

